# Cumulative Exposure and Health Risk Assessment of PFAS in Animal-Derived Foods Using the Relative Potency Factor Approach

**DOI:** 10.3390/toxics13110931

**Published:** 2025-10-30

**Authors:** Giulia Rampazzo, Francesco Arioli, Giampiero Pagliuca, Giacomo Depau, Elisa Zironi, Teresa Gazzotti

**Affiliations:** 1 Department of Veterinary Medical Sciences, Alma Mater Studiorum University of Bologna, 40064 Ozzano dell’Emilia, Italy; giulia.rampazzo4@unibo.it (G.R.); giacomo.depau2@unibo.it (G.D.); elisa.zironi@unibo.it (E.Z.); teresa.gazzotti@unibo.it (T.G.); 2 Department of Veterinary Medicine and Animal Sciences, University of Milan, 26900 Lodi, Italy; francesco.arioli@unimi.it

**Keywords:** risk assessment, PFAS, RPF, animal-derived food, food safety, consumer exposure

## Abstract

Per- and polyfluoroalkyl substances (PFASs) are persistent and bioaccumulative contaminants frequently detected in animal-derived foods, raising concerns for consumer health. In 2020, the European Food Safety Authority (EFSA) established a group tolerable weekly intake (TWI) of 4.4 ng/kg bw per week for four PFAS (PFOA, PFNA, PFOS, PFHxS) based on immunotoxicity, prompting the European Commission to set maximum levels in food. However, many other PFAS are present in the diet, and their cumulative risk is poorly characterized. This study applied the Relative Potency Factor (RPF) approach, using hepatic toxicity as the reference endpoint. The RPF approach addresses a key challenge in assessing human dietary exposure to PFAS by enabling cumulative risk assessment for complex mixtures found in food, moving beyond single-compound evaluations. Occurrence data from EFSA’s 2020 opinion were combined with European consumption data for fish, meat, eggs, and milk across four population groups (toddlers, adolescents, adults, elderly). Exposure estimates, expressed in PFOA equivalents, were compared with the group TWI. Results showed toddlers as the most vulnerable, with cumulative exposure approaching or exceeding TWI through fish, meat, and eggs, while milk contributed less. PFOS and PFOA were the main contributors across all food categories, with PFNA and PFDA also relevant, especially in younger populations. The findings highlight the added value of the RPF approach for cumulative PFAS risk assessment and emphasize the need for updated monitoring, refinement of potency factors for under-studied PFAS, and continued regulatory measures to protect high-risk consumers.

## 1. Introduction

Per- and polyfluoroalkyl substances (PFASs) are a large class of synthetic compounds widely used for their surfactant properties and chemical stability. Their persistence and bioaccumulative potential have led to widespread environmental and food contamination. PFAS are often found as mixtures in environmental and food matrices, leading to combined human exposure [1,2,3,4,5]. Dietary intake is the primary route of exposure for the general population, which significantly contribute to internal PFAS accumulation [2].

In response to growing toxicological and epidemiological evidence, the European Food Safety Authority (EFSA) has established health-based guidance values (HBGVs) for selected PFAS. In 2020, EFSA set a group tolerable weekly intake (TWI) of 4.4 ng/kg body weight per week for PFOA, PFNA, PFOS, and PFHxS, based on immunotoxicity, specifically reduced vaccine antibody responses in children, a sensitive endpoint [2]. Although HBGVs are typically derived from comprehensive toxicological investigations in humans or animals, such datasets are currently available for only a limited number of substances, primarily PFOA and PFOS [2,6]. Nevertheless, PFNA and PFHxS were included in the group TWI based on their concordant toxicokinetic characteristics, comparable toxicological responses in animal studies, overlapping human biomonitoring data, and consistent occurrence patterns in contamination studies [2]. Building on these risk assessment outcomes, the European Commission has established Maximum Levels (MLs) in food for PFOA, PFOS, PFNA, PFHxS, and their sum [7], although these compounds represent only a small subset of the more than 4700 PFAS identified by the Organisation for Economic Co-operation and Development (OECD) in 2021 [8].

Several PFASs have been associated with negative effects on human health, such as immunotoxicity, hepatotoxicity, developmental toxicity, and metabolic disorders. Moreover, in 2023, the IARC classified PFOA as carcinogenic to humans (Group 1) and PFOS as possibly carcinogenic (Group 2B) [9].

To enhance chemical safety assessment while reducing reliance on animal testing, European agencies, including EFSA and ECHA, are promoting New Approach Methodologies (NAMs), such as grouping and read-across strategies. These methods extrapolate data from well-characterized substances to structurally or toxicologically related compounds, applying criteria based on shared structural features, toxicokinetic profiles, or modes of action. Currently, EFSA is developing guidance for implementing read-across within food and feed risk assessment frameworks [10].

Although awareness of the health risks linked to PFAS is increasing, major obstacles persist in accurately measuring cumulative human dietary exposure due to the variety of PFAS mixtures found in foods. Conventional risk assessments have generally targeted individual compounds like PFOS or PFOA, but environmental and food samples often contain complex PFAS mixtures, requiring novel methods capable of assessing total toxicity more effectively. The Relative Potency Factor (RPF) approach may fill this gap by allowing the inclusion of sum parameters in risk evaluations, thereby providing a more realistic representation of actual exposure conditions. This approach is consistent with the REACH Regulation [11], which advocates the use of non-testing methods wherever possible.

In mixture risk assessment, the dose-addition principle is commonly used to estimate the combined toxicity of structurally related chemicals with similar effects, even when acting through different mechanisms [12,13]. EFSA applies this approach for pesticide residues and recommends the RPF method to quantify the toxicity of mixture [14]. The RPF method, analogous to the Toxic Equivalency Factor (TEF) approach used for dioxins, scales the potency of each compound relative to an Index Compound (IC, typically, =1), enabling cumulative exposure estimation in IC equivalents. The estimated cumulative exposure is then compared with the HBGV of the IC, such as a TWI. The RPF approach relies on a well-defined toxicological endpoint, ideally derived from repeated-dose toxicity studies [13,14]. Bil et al. [15] assessed the risk associated with oral exposure to 23 PFAS by using liver hypertrophy and increased liver weight in male rats as common toxicological endpoints, developing specific relative RPFs accordingly. Each compound’s RPF was calculated by comparing its benchmark dose (BMD) to that of a predefined IC (PFOA), which was assigned an RPF value of 1. The BMD is the estimated dose of a substance that causes a minimal but statistically significant biological effect (e.g., increase in liver weight) compared to an unexposed control group.

This approach enabled the translation of external doses or measured concentrations of individual PFAS into PFOA equivalents (PEQ). This set of RPFs is useful when estimating the risk resulting from mixtures of PFAS in, for instance, drinking water or food samples [15].

In this context, regulatory agencies are increasingly prioritizing the monitoring of chemical mixtures and the application of RPFs to improve the assessment of combined effects. In its 2020 opinion, EFSA also strongly recommended further studies to support the derivation of potency factors for PFAS [2].

Notably, the 2023 Drinking Water Directive [16] was the first to introduce the concepts of “PFAS sum” (sum of PFAS considered a concern as regards water intended for human consumption according to the directive) and “Total PFAS” (the totality of per- and polyfluoroalkyl substances). It expanded the list of monitored PFAS compounds to 24, while also emphasizing the importance of monitoring as many PFASs as possible. Furthermore, the European Commission has adopted a similar approach in the new directive for surface waters. The Commission’s proposal now includes the RPF approach for PFAS assessment, whereby the sum Environmental Quality Standard (EQS) is expressed in terms of PEQ [17]. The U.S. approach to PFAS mixtures in food relies on EPA’s toxicity values to enable the FDA’s risk assessments, which employ tools like the Hazard Index to evaluate the cumulative risk from multiple PFAS detected in food items [18]. The fundamental principle behind the Hazard Index is that chemicals with similar toxic effects (e.g., all causing liver damage) have additive effects. This means the total risk is the sum of the risks posed by each individual chemical in the mixture [19].

Considering recent regulatory developments and growing scientific evidence, the need to evaluate as many PFAS substances as possible in food, along with their real implications for consumer health, is evident. This study aimed to assess the risk of European consumers’ exposure to PFAS through the consumption of animal-derived food products expressed as PEQ, with a specific focus on hepatic toxicity as the target endpoint. This was achieved by applying the RPF approach, which represents a promising tool to refine PFAS risk assessment. However, it is important to clarify that the RPF protocol developed by Bil et al. [15], is not yet officially recognised or validated at the European level. Therefore, some degree of uncertainty—including potential overestimation of exposure—cannot be excluded. Further validation is still needed before its routine implementation in regulatory frameworks.

## 2. Materials and Methods

### 2.1. PFAS Occurrence, Consumption, and Consumer Data

This study was conducted using food occurrence data on PFAS provided in the 2020 EFSA scientific opinion (Annex A) on the risk to human health related to the presence of PFAS in food. Specifically, PFAS occurrence data for the following food categories (FCs) were considered: fish and sea products, meat and meat products (including edible offal), eggs and egg products, and milk and dairy products. For this work, the mean lower bound (LB) occurrence values were used for all four FCs [20]. Mean LB occurrence values were selected to provide a conservative but realistic estimate of exposure, consistent with the approach recommended by the CONTAM Panel in the EFSA 2020 opinion [2]. The occurrence data were combined with food consumption data retrieved from the EFSA Comprehensive European Food Consumption Database, which provides detailed consumption information for different population groups across Europe. Consumption data for the four FCs were extracted for four specific age groups of consumers: toddlers, adolescents, adults, and the elderly. An average European consumption value was calculated as a weighted mean of the individual Member State surveys, for each FC and consumer group (Table 1). Food consumption patterns differ across Europe (e.g., higher fish intake in Northern vs. Southern countries), and the use of weighted average data may mask such regional variability. Therefore, while our approach provides a European-level overview, it is important to consider that real-life dietary differences could lead to regional disparities in PFAS exposure and related risks.

Finally, standard body weights were applied for each consumer group, as recommended by EFSA: 12 kg for toddlers, 45 kg for adolescents, and 70 kg for adults and the elderly [21].

### 2.2. PEQ Exposure Assessment and Characterization

To estimate consumer exposure to PFAS the Estimated Weekly Intake (EWI) for each PFAS compound was calculated by combining occurrence data with average food consumption and standard body weight values for each food category (FC) and consumer group. The concentrations of individual PFAS were converted into PEQ by applying RPFs, which reflect the toxicity of each compound relative to PFOA. For certain PFAS, RPFs were estimated using a read-across approach and expressed as a range (Table 2), based on the assumption that their potency lies between that of shorter- and longer-chain perfluoroalkyl carboxylic or sulfonic acids [15]. For these compounds, exposure was calculated using both the minimum and maximum RPF values.

The following Equation (1) represents the cumulative EWI for a given FC, obtained by summing the contributions of all PFAS present in that category, each weighted by its RPF and adjusted for consumption and body weight:

(1) $$\mathrm{PEQ}\text{-}\mathrm{Cumulative}\;{\mathrm{EWI}}_{\mathrm{FC}}\;=\;\sum_{i=1}^{n}\frac{{Concentration}_{i}\times {RPF}_{i}(min/max)\times Mean\;consumption}{BW}\times 7$$

Concentration_i_ = concentration of PFAS_i_ in the food category (ng/kg)

RPF_i_ = relative potency factor of PFAS_i_

Mean Consumption = average daily consumption of the food category (kg/day)

BW = standard body weight for the consumer group (kg)

7 = conversion from daily to weekly intake

The calculated individual PFAS PEQ-EWI and the PEQ cumulative EWI for each FC and consumer group were compared to the HBGV (TWI: 4.4 ng/kg bw per week) to obtain the %TWI. To assess the differences in %TWI contribution, PEQ-EWIs were compared with those obtained through the traditional concentration-based method (CB-EWIs) (2) (Complete dataset is provided in the Supplementary Materials).

(2) $$\mathrm{CB}\text{-}\mathrm{Cumulative}\;{\mathrm{EWI}}_{\mathrm{FC}}\;=\sum_{i=1}^{n}\frac{{Concentration}_{i}\times Mean\;consumption}{BW}\times 7$$

Dietary exposure (PEQ-Total EWI or CB-Total EWI), for each consumer group, was estimated by summing the cumulative exposures from the four selected animal-derived FC for both PEQ and CB methods (3).

(3) $$\mathrm{PEQ}\;\mathrm{or}\;\mathrm{CB}\text{-}\mathrm{Total}\;\mathrm{EWI}=\sum_{j=1}^{n}PEQ\;or\;CB\;Cumulative\;{EWI}_{\mathrm{FCj}}$$

The Hazard Index (HI) for each consumer group was calculated following EFSA guidelines [22] (4):

(4) HI=HQ

where the $HQ=Hazard\;Quotient=\frac{PEQ\;Total\;EWI\;or\;CB\;Total\;EWI}{TWI}$ calculated for each consumer group.

### 2.3. Data Presentation

Numerical results are reported up to two decimal places. Values reported as ‘0.00’ in the tables should not be interpreted as absent. They represent concentrations or exposure levels that are greater than zero but below the precision of the second decimal place.

In this study, the results obtained using the RPF approach were compared with those derived from a traditional CB risk assessment approach. It should be noted that both sets of exposure estimates were calculated by the authors, based on EFSA’s publicly available occurrence and food consumption datasets, rather than directly taken from the exposure values reported in the EFSA opinion. Consequently, the exposure estimates presented here are not directly comparable to those reported by EFSA but were generated to enable a consistent comparison between the two approaches under a harmonised dataset. The estimated exposures obtained in this study were compared to the TWI (for details, consult the Supplementary Materials). Although the current TWI is established exclusively for PFOA, PFOS, PFNA, and PFHxS, it was used as the reference value, as it represents the official, sole, and up-to-date HBGV currently available in Europe. It is important to note that the TWI established by EFSA was based on immunotoxicity as the critical endpoint, whereas the RPFs used in this study refer to hepatic toxicity; therefore, the data are not directly comparable. However, since the 2020 EFSA opinion stated that the TWI also protects against other potential adverse effects in humans [2], the comparison was still included. Moreover, as mentioned in the Introduction section, this methodological distinction highlights the exploratory nature of the present work and the need for further validation of the RPF approach before its broader application in routine risk assessment.

Therefore, although the EFSA assessment covered 26 PFAS and RPFs were developed for 23, this study focused only on PFAS for which both RPFs and occurrence data were available, which explains the smaller number of compounds reported in Table 2.

## 3. Results and Discussion

### 3.1. Considerations on PFAS Occurrence Data

In the EFSA scientific opinion on PFAS in food, the CONTAM Panel reported a final dataset of 69,433 results covering 26 PFAS. Importantly, 92% of these results were left-censored (below the limit of detection [LOD] or limit of quantification [LOQ]). The Panel noted that, for most food categories and PFAS, mean upper bound (UB) levels—calculated by assigning LOD/LOQ values to non-detects—were much higher than mean LB levels—calculated by assigning zero to non-detects. This large discrepancy was mainly due to the high proportion of left-censored data and/or the limited availability of data for many PFAS. Consequently, exposure estimates were considered only as a rough indication of the potential range of chronic dietary exposure and should be interpreted with caution. Based on these considerations, the CONTAM Panel concluded that LB exposure estimates are more realistic than UB estimates. It should be emphasized, however, that the fact that 92% of food samples did not contain quantifiable PFAS is highly relevant from a repeated-exposure perspective: it implies that in most food consumption events, dietary intake of PFAS is negligible or absent. In our study, to compare the RPF approach for risk characterisation with the assessment carried out by EFSA, PFAS occurrence data were used as recommended by the CONTAM panel [2]. It can be assumed that the average values used for risk estimation were largely determined by outliers with high PFAS concentrations, a part of the small 8% of quantifiable samples, but which have a significant impact on the calculated average exposure, mainly reflecting the actual contribution of highly contaminated foods. The occurrence data on PFAS in food used in this study were extracted from the EFSA opinion and refer to samples collected up to 2018. Since then, contamination patterns may have shifted, both in terms of overall concentrations and in the relative contribution of legacy versus emerging PFAS. In parallel, analytical methodologies for PFAS determination continue to advance, enhancing sensitivity and expanding the range of detectable compounds. These developments highlight the urgent need for updated and harmonized monitoring data at both the European and global levels to ensure accurate and reliable consumer risk assessment. Although this study is based on official monitoring data, further progress in PFAS analysis largely depends on the continuous improvement of detection and extraction techniques developed by research laboratories. To ensure comparability and reliability of data generated through such diverse analytical approaches, the adoption of common benchmarks is essential. In this context, the performance criteria established by Regulation (EU) 2022/1431 [23] and the guidelines provided by the European Union Reference Laboratory for Halogenated Persistent Organic Pollutants in Feed and Food (EURL POPs) [24] play a key role in standardizing data quality across laboratories by defining consistent requirements for recovery, precision, and sensitivity.

### 3.2. Evaluation of PFAS Intake Through Consumption of Milk and Dairy Products

The EWI of PFAS, expressed as PEQ, via consumption of milk and dairy products, was assessed for four population groups under the LB scenario (Table 3). Cumulative PEQ exposure ranged from 0.07–0.08 ng/kg bw per week in adults and the elderly, 0.14–0.15 ng/kg bw per week in adolescents, and 0.54–0.59 ng/kg bw per week in toddlers. In all groups, cumulative exposure remained well below the HBGV. The highest exposure was observed in toddlers, accounting for up to 13% of the TWI under the maximum exposure scenario, whereas adolescents, adults, and the elderly reached 3%, 2%, and 2%, respectively. Among individual PFAS, PFOS was the primary contributor to overall exposure across all groups, particularly in toddlers (0.29 ng/kg bw per week), followed by PFOA (0.12 ng/kg bw per week). PFNA (0.07 ng/kg bw/week) and PFDA (0.03–0.08 ng/kg bw per week) contributed modestly but non-negligibly, especially in toddlers, where they accounted for up to 2–3% of the TWI. Other compounds, including PFBS, PFHxA, PFHxS, and PFPeA, contributed minimally (<0.01 ng/kg bw per week in all groups).

This distribution reflects the environmental persistence and bioaccumulation potential of long-chain PFAS [25]. Up-to-date monitoring data consistently identify PFOS as the predominant contaminant in milk, followed by PFOA [26]. For short-chain PFAS such as PFBS and PFHxA, estimated exposure was negligible, consistent with their low bioaccumulation and rapid excretion described in previous studies [25]. It is noteworthy that most investigations on PFAS in milk focus on cow’s milk, whereas data on non-cow milk remain scarce. In the limited studies available, PFOS consistently emerged as the most frequently detected PFAS, also in other species, with PFTeDA also among the predominant compounds [22]. This research gap constrains the understanding of exposure and limits the ability to conduct accurate risk assessments for populations consuming non-bovine milk.

When comparing PEQ estimated exposures with estimates derived using the traditional CB approach (values indicated with *), cumulative EWI values obtained through the RPF-based approach were consistently slightly lower across all population groups, primarily due to the relative contribution of PFPeA. CBapproach indicated a substantial contribution of PFPeA (up to 26% of the TWI in toddlers), which was not reflected in the RPF-based estimates due to the low hepatic toxicity reference point assigned to this compound (RPF = 0.03). This discrepancy suggests that the CB approach may overestimate the risk of hepatic toxicity from PFPeA in milk and dairy products compared with the RPF-based method. At the same time, the RPF-based approach highlights a modest contribution of PFDA to toddlers’ exposure, the population group identified as most at risk. PFDA remains a comparatively less studied compound; its RPF value, ranging between 4 and 10 based on read across, indicates a moderate potential impact on hepatic toxicity, underscoring the need for further investigation, especially for younger consumers.

### 3.3. Evaluation of PFAS Intake Through Consumption of Meat and Meat Products

For meat and meat products (including animal offal), Table 4 summarizes the EWI PEQ across the different consumer groups. The highest cumulative exposure was observed in toddlers, ranging from 4.11 to 4.44 ng/kg bw per week, corresponding to 93–101% of the TWI, which is concerning. In this group, PFOS was the predominant contributor (58% of the TWI), followed by PFOA (13%), PFDA (up to 10%), PFNA (7%), and PFDoDA (7%).

Among adolescents, cumulative PEQ intake ranged from 2.91 to 3.15 ng/kg bw per week, representing 66–72% of the TWI, with PFOS again dominating the exposure profile (41%), followed by PFOA (9%) and PFDA (up to 7%). Adults and elderly consumers exhibited lower cumulative exposures (2.04–2.20 ng/kg bw per week and 1.77–1.91 ng/kg bw per week, respectively), equivalent to 46–50% and 40–43% of the TWI. Across all age groups, PFOS and PFOA consistently emerged as the main contributors, while PFDA, PFDoDA, and PFUnDA accounted for smaller but non-negligible shares (1–10%). Most of the remaining PFAS (e.g., PFBS, PFHpA, PFHxA, PFPeA, PFTrDA) contributed negligibly to all groups. Toddlers and adolescents were confirmed as the most exposed and, therefore, the most vulnerable consumer categories. PFOS emerged as the most frequently detected PFAS in meat and meat products, especially when animal offal was included, reflecting its tendency to accumulate in organs and tissues such as the liver, kidneys, and spleen [27,28]. Evidence further suggests that PFAS concentrations are generally lower in farmed animals compared with game species, pointing to distinct environmental exposure pathways [29]. For the four regulated PFAS (PFOS, PFOA, PFNA, PFHxS), concentrations typically follow the order wild boar > bovine > deer > pig > chicken [29]. Overall, these findings highlight the added value of the RPF approach in capturing cumulative PFAS exposure and emphasize the importance of integrating dietary habits and regional consumption patterns into refined, health-protective risk assessments.

### 3.4. Evaluation of PFAS Intake Through Consumption of Eggs and Egg Products

Table 5 reports the EWI of PEQ through eggs and egg products consumption across consumer groups. Among all age groups, toddlers again showed the highest cumulative intake, with values ranging from 3.80 to 3.85 ng/kg bw per week, corresponding to 86–88% TWI. The main contributors to total PEQ intake from eggs and egg products in toddlers were PFOS (72% of the TWI) and PFOA (14%), while PFTrDA, PFHpA, PFHxA, PFHxS, and PFUnDA contributed marginally or were negligible.

Adolescents, adults, and the elderly showed lower cumulative intakes, ranging from 1.16 to 1.63 ng/kg bw per week, representing 26–37% of the TWI. In all age groups, PFOA and PFOS were consistently the predominant contributors to total exposure. The intake of other PFAS was very low or non-detectable, with contributions below 1% of the TWI. When comparing the two exposure assessment approaches, only minor differences were observed for PFAS intake through eggs and egg products. The main exception was PFOS, for which the RPF-based method indicated a substantially greater contribution to reaching the HBGV for hepatic toxicity, suggesting that the traditional CB approach may underestimate risk, particularly for younger consumers who appear to be the most vulnerable.

PFAS concentrations in eggs are influenced by a combination of environmental factors, farming practices, and dietary inputs. Eggs produced in proximity to industrial sources or under free-range systems frequently show elevated levels, particularly of PFOS and other long-chain PFAS such as PFOA, PFHxS, and PFNA, which display a strong affinity for egg lipoproteins and consequently accumulate efficiently [30,31]. Feed composition, including the use of contaminated water or fishmeal, constitutes a major exposure pathway, while the ingestion of soil and dust likely explains the higher PFAS concentrations often reported in home-produced and free-range eggs compared to commercial ones [30,32]. Additional variables, such as hen age and laying performance, may further modulate PFAS transfer into eggs [33]. Overall, these findings underscore the multifactorial nature of PFAS contamination in eggs and highlight the need to account for both production systems and environmental conditions when assessing dietary exposure.

### 3.5. Evaluation of PFAS Intake Through Consumption of Fish and Seafood

Table 6 summarizes the EWI of PFAS, expressed as PEQ, from fish and seafood consumption across different consumer groups, showing a pronounced age-related trend. Toddlers exhibited the highest cumulative exposure, with values ranging from 22.62 to 25.93 ng/kg bw per week, exceeding the TWI by 514–589%. This excessive intake was primarily driven by PFOS, which alone accounted for 354% of the TWI, followed by PFNA (58%), PFDA (44–110%), PFOA (31%), and PFUnDA (17%). Adolescents, adults, and the elderly also showed substantial exceedances, with cumulative EWIs ranging from 12.06 to 14.58 ng/kg bw per week (274–331% of the TWI). In these groups, PFOS remained the dominant contributor (175–199% of the TWI), with relevant inputs from PFNA (31–33%), PFOA (29–33%), PFDA (23–62%), and minor contributions from PFUnDA (up to 9%) and PFTrDA (≤5%).

When comparing these results with those obtained using the CB approach, cumulative EWIs estimated with the RPF-based method were consistently higher across all age groups. Nevertheless, it is noteworthy that both approaches indicated exposures exceeding the HBGV. PFOS was the main driver in all scenarios, reinforcing its central role in dietary PFAS risk. As observed for other food categories, toddlers and adolescents emerged as the most exposed and, therefore, the most vulnerable population groups.

The RPF-based assessment also highlighted the contribution of additional long-chain PFAS, particularly PFDA and PFUnDA, which were underestimated using the classical CB approach. For example, in toddlers, PFDA contributed 44–110% of the TWI under the RPF approach compared with only 0–11% using the CB methodology. This discrepancy suggests that the classical approach may undervalue the risk posed by PFAS with high toxic potency but present at lower absolute concentrations. Conversely, short-chain PFAS such as PFBS, PFDS, PFHpA, and PFHxA contributed negligibly in both approaches, confirming their limited relevance in the overall dietary risk from fish and seafood.

Compounds such as PFOS, PFOA, and PFDA accumulate more efficiently in aquatic food webs than short-chain PFAS (e.g., PFBS, PFBA) due to their physicochemical properties and strong affinities for sediments and benthic organisms [28,34]. In this context, species-specific differences and trophic magnification are key drivers, with higher trophic level piscivorous fish and sediment-ingesting detritivores, such as crustaceans, showing particularly high concentrations [28,34]. Tissue distribution further modulates exposure, as PFAS tend to accumulate in organs such as the liver, while muscle tissue generally exhibits lower levels [27,34,35]. Geographic origin also plays a critical role: fish from areas affected by urban and industrial effluents, including wastewater discharges and landfill leachates, frequently display elevated PFAS burdens [36,37,38]. Conversely, farmed fish generally show lower contamination compared to their wild-caught counterparts [39,40]. Taken together, these findings highlight the complex interplay of biological, ecological, and environmental factors governing PFAS accumulation in aquatic food webs and underscore the importance of considering species, tissue type, and geographic origin when assessing dietary exposure.

### 3.6. Dietary Exposure Assessment and Characterization Through Animal-Derived Foods Across Consumer Groups

Table 7 shows the dietary exposure assessment considering all the FC for all the consumer groups and the derived HI. The HI is a harmonized approach commonly applied in the risk characterization of combined exposure to multiple chemicals acting on the same organ or system, particularly for non-carcinogenic effects [41]. A HI < 1 is generally interpreted as indicating low concern or no concern for human health. Conversely, a HI > 1 may suggest the need for a refined risk assessment or the consideration of risk management measures [41]. Among the four groups, toddlers exhibited the highest total dietary PFAS exposure, with cumulative dietary EWI values ranging from 31.07 to 34.81 ng/kg bw per week. This corresponded to HI ranging from 7.06 to 7.91, while the CB-HI reached 3.98, both well above the health-based threshold of 1. The higher HI values obtained with the PEQ-HI highlight how the RPF weighting, based on hepatic toxicity, provides a potentially more conservative estimate compared with the CB approach. The dominant source of exposure in this group was fish and seafood, which alone accounted for up to 22.62 and 25.93 ng/kg bw per week. Other notable contributors included eggs and egg products and meat and meat products, while milk and dairy products had a relatively minor impact.

In adolescents, the total dietary EWI ranged between 17.40 and 19.50 ng/kg bw per week, resulting in PEQ-HI values of 3.95–4.43 and a CB-HI of 2.15. Fish and seafood remained the primary contributors to exposure, followed by meat and eggs. Adults showed a total dietary EWI of 14.54 to 16.35 ng/kg bw per week, corresponding to PEQ-HI values of 3.30–3.72 and a CB-HI of 1.76. In this group as well, fish and seafood represented the largest exposure source, followed by meat and eggs. Similarly, the elderly population showed comparable exposure levels to adults, with a total dietary EWI ranging from 15.06 to 16.99 ng/kg bw per week and PEQ-HI values of 3.42–3.86, and a CB-HI of 1.82. Once again, fish and seafood accounted for the majority of intake, followed by meat and eggs. In all consumer groups, the PEQ-HI threshold of 1 was exceeded by at least threefold, reaching up to sevenfold in the worst-case scenario for toddlers. These results may point to a potential health concern, particularly for younger consumers, and underscore the need for a more refined risk assessment and, if confirmed, the consideration of appropriate risk management measures.

The comparison between PEQ-HI and CB-HI values underscores the added potential value of the RPF-based approach, which integrates compound-specific toxic potency into the cumulative assessment. While the CB-HI provides a useful first-tier indication of combined exposure based solely on concentrations, the PEQ-HI refines the risk estimate by accounting for inter-compound toxicity differences, resulting in a more precautionary and health-protective outcome.

These findings, consistent with the conclusions of the EFSA 2020 opinion, identified fish and other seafood as the primary contributors to PFOS and PFOA exposure, followed by meat and meat products and eggs and egg products Our results indicate that, when PFAS exposure is assessed using specific RPF with hepatic toxicity as the critical endpoint, the European population may exceed the TWI through the consumption of animal-derived products alone, raising a significant cause for concern. Moreover, EFSA also emphasized that PFAS exposure is not limited to animal-based foods: fruit and fruit products, starchy roots and tubers were identified as important contributors to combined exposure, while for PFOA, vegetables, vegetable products, and drinking water represented significant sources. Overall, the RPF-based approach demonstrated its usefulness in refining cumulative PFAS risk assessment. However, as this methodology has not yet been officially validated at the European level, further evaluation and validation efforts are needed.

### 3.7. Future Perspectives

Current toxicological knowledge for PFAS is restricted to a few well-studied compounds, primarily PFOA and PFOS, with considerably less information available for PFNA, PFHxS, and almost no in vivo data for the vast majority of emerging PFAS [42]. This scarcity of toxicological evidence significantly hinders the derivation of relative RPFs across multiple endpoints, limiting the ability to conduct robust cumulative risk assessments, as also highlighted by EFSA (opinion 2020). In this context, the prediction and evaluation of toxicity after compound exposure has become one of the core research topics of PFAS [42] and provides enormous research space for the application of Machine Learning (ML). ML and Quantitative Structure–Activity Relationship (QSAR) modelling represent promising approaches to address this gap by integrating chemical structure, physicochemical descriptors, and available bioactivity or in vitro data to estimate toxic potency for data-poor PFAS [43]. By generating provisional toxicological thresholds and corresponding RPFs for a broad range of endpoints, these computational tools could significantly improve the accuracy of dietary exposure assessments to PFAS, facilitate the identification of high-priority compounds for regulatory monitoring, and guide the design of targeted in vivo studies focusing on critical outcomes such as developmental or immunological effects.

However, the application of ML and QSAR to PFAS toxicity evaluation remains in its early stages and faces significant challenges. Current models are limited by the small number of PFAS with reliable in vivo dose–response data for training, the complex and often non-linear toxicokinetic of PFAS, and the need to integrate mechanistic information to improve prediction accuracy [43]. To fully realize their potential, future efforts should prioritize expanding standardized toxicity datasets, integrating computational methods with adverse outcome pathway (AOP) frameworks, and establishing harmonized regulatory guidance and standards for the use of ML- and QSAR-based models [43], including the use of RPFs in risk assessment. Such standardization is essential to ensure that results are comparable, transparent, and suitable for regulatory application. These advances would enable ML and QSAR tools to become integral components of cumulative PFAS risk characterization, thereby supporting evidence-based regulatory decisions and enhancing public health protection.

## 4. Conclusions

This study assessed dietary exposure to PFAS from animal-derived foods across European populations using the RPF approach, with hepatic toxicity as the critical endpoint. Fish and seafood were the main contributors, followed by meat and eggs, while milk and dairy products played a minor role. Exposure was largely driven by PFOS and other long-chain PFAS (PFNA, PFDA, PFOA), and both CB and RPF-based assessments showed that fish and seafood alone can exceed the TWI. When all animal-derived foods were considered with the RPF approach, intakes surpassed the HBGV (HI > 1), indicating potential hepatic risks, particularly in toddlers, who consistently showed the highest exposures. In the EFSA assessment, 92% of samples were left-censored, implying that exposure stems from only ~8% of quantifiable samples. Consequently, mean LB values were strongly influenced by a few high-concentration outliers. Our findings confirm that these occasional but highly contaminated foods disproportionately shape average exposure estimates, highlighting their real contribution to dietary risk.

The RPF approach allowed a more mechanistic characterization of risk, revealing that PFDA, PFDoDA, and PFUnDA may contribute more significantly to liver toxicity than previously recognized, while traditional method may overestimate risk from PFPeA in dairy products. In such a dynamic context, where both legacy and emerging PFAS coexist in the food chain, RPFs may provide a practical tool for more rapid risk assessment, as they allow hazard estimation without the need to wait for individual dose–response studies for each PFAS. Overall, these results underscore the need for continued monitoring, regulatory evaluation, and targeted risk management, particularly for younger consumers. They also suggest that current TWI values may not fully protect the most vulnerable groups. Future research may integrate computational tools such as machine learning, QSAR modelling, and mechanistic frameworks (e.g., adverse outcome pathways) to refine RPFs across multiple endpoints. Such advancements will improve the accuracy and regulatory relevance of PFAS risk assessments, ultimately strengthening public health protection. However, it is essential to emphasize that the RPF approach has not yet been officially validated at the European level, and further methodological harmonisation and experimental validation are needed before it can be routinely applied in regulatory risk assessment.

## Figures and Tables

**Table 1:** European mean consumption for each animal-derived FC and consumer group.

Food Category	Consumer Group	G/Day
Fish and seafood	Toddlers	9.4
	Adolescents	18.7
	Adults	25.6
	Elderly	28.9
Meat and meat products	Toddlers	47.0
	Adolescents	125.3
	Adults	135.9
	Elderly	117.7
Eggs and egg products	Toddlers	10.1
	Adolescents	15.9
	Adults	18.6
	Elderly	18.3
Milk and dairy products	Toddlers	324.5
	Adolescents	318.0
	Adults	254.6
	Elderly	237.1

**Table 2:** RPFs applied based on liver hypertrophy as a toxicological endpoint [15].

Compound	RPF	
	Min	Max
PFBA	0.1	
PFBS	0	
PFDA	4	10
PFDS	2	
PFDoDA	3	
PFHpA	0	1
PFHpS *	0.6	2
PFPeS *	0.001	0.6
PFHxA	0	
PFHxS	0.6	
PFHxDA	0.02	
PFNA	10	
PFOA	1	
PFOS	2	
PFODA	0.02	
PFPeA	0	
PFTeDA	0.3	
PFTrDA *	0.3	3
PFUnDA	4	

* Based on read across [15].

**Table 3:** PEQ estimated intakes through Milk and milk products consumption ng/kg bw per week.

		PFBS	PFDA (Min)	PFDA (Max)	PFHxA	PFHxS	PFNA	PFOA	PFOS	PFPeA	Cumulative EWI (Min)	Cumulative EWI (Max)
Toddlers	PEQ-EWI	0.00	0.03	0.08	0.00	0.00	0.07	0.12	0.29	0.03	0.54	0.59
	%TWI	0%	1%	2%	0%	0%	2%	3%	7%	1%	12%	13%
	CB-%TWI *	0%	0%		0%	0%	0%	3%	3%	26%	33%	
Adolescents	PEQ-EWI	0.00	0.01	0.02	0.00	0.00	0.02	0.03	0.08	0.01	0.14	0.15
	%TWI	0%	0%	0%	0%	0%	0%	1%	2%	0%	3%	3%
	CB-%TWI *	0%	0%		0%	0%	0%	1%	1%	7%	9%	
Adults	PEQ-EWI	0.00	0.00	0.01	0.00	0.00	0.01	0.02	0.04	0.00	0.07	0.08
	%TWI	0%	0%	0%	0%	0%	0%	0%	1%	0%	2%	2%
	CB-%TWI *	0%	0%		0%	0%	0%	0%	0%	4%	4%	
Elderly	PEQ-EWI	0.00	0.00	0.01	0.00	0.00	0.01	0.01	0.04	0.00	0.07	0.07
	%TWI	0%	0%	0%	0%	0%	0%	0%	1%	0%	2%	2%
	CB-%TWI *	0%	0%		0%	0%	0%	0%	0%	3%	3%	

* Value representing the % ratio between CB-EWI and the TWI.

**Table 4:** PEQ estimated intakes through meat and meat products consumption ng/kg bw per week.

Consumer Group		PFBA	PFBS	PFDA (Min)	PFDA (Max)	PFDS	PFDoDA	PFHpA (Min)	PFHpA (Max)	PFHpS (Min)	PFHpS (Max)	PFHxA	PFHxS	PFNA	PFOA	PFOS	PFPeA	PFTeDA	PFTrDA (Min)	PFTrDA (Max)	PFUnDA	Cumulative EWI (Min)	Cumulative EWI (Max)
Toddlers	PEQ-EWI	0.00	0.00	0.18	0.44	0.09	0.32	0.00	0.02	0.00	0.01	0.00	0.00	0.29	0.57	2.53	0.00	0.00	0.01	0.05	0.11	4.11	4.44
	%TWI	0%	0%	4%	10%	2%	7%	0%	0%	0%	0%	0%	0%	7%	13%	58%	0%	0%	0%	1%	2%	93%	101%
	CB-%TWI *	2%	0%	1%		1%	2%	0%		0%		1%	0%	1%	13%	29%	3%	0%	0%		1%	54%	
Adolescents	PEQ-EWI	0.00	0.00	0.12	0.31	0.06	0.23	0.00	0.01	0.00	0.00	0.00	0.00	0.21	0.4	1.8	0.00	0.00	0.00	0.04	0.07	2.91	3.15
	%TWI	0%	0%	3%	7%	1%	5%	0%	0%	0%	0%	0%	0%	5%	9%	41%	0%	0%	0%	1%	2%	66%	72%
	CB-%TWI *	1%	0%	1%		1%	2%	0%		0%		1%	0%	0%	9%	20%	2%	0%	0%		0%	39%	
Adults	PEQ-EWI	0.00	0.00	0.09	0.22	0.04	0.16	0.00	0.01	0.00	0.00	0.00	0.00	0.14	0.28	1.26	0.00	0.00	0.00	0.03	0.05	2.04	2.20
	%TWI	0%	0%	2%	5%	1%	4%	0%	0%	0%	0%	0%	0%	3%	6%	29%	0%	0%	0%	1%	1%	46%	50%
	CB-%TWI *	1%	0%	0%		0%	1%	0%		0%		1%	0%	0%	6%	14%	2%	0%	0%		0%	27%	
Elderly	PEQ-EWI	0.00	0.00	0.08	0.19	0.04	0.14	0.00	0.01	0.00	0.00	0.00	0.00	0.12	0.25	1.09	0.00	0.00	0.00	0.02	0.05	1.77	1.91
	%TWI	0%	0%	2%	4%	1%	3%	0%	0%	0%	0%	0%	0%	3%	6%	25%	0%	0%	0%	1%	1%	40%	43%
	CB-%TWI *	1%	0%	0%		0%	1%	0%		0%		0%	0%	0%	6%	12%	0%	0%	0%		0%	23%	

* Value representing the % ratio between CB-EWI and the TWI.

**Table 5:** PEQ estimated intakes through eggs and egg products consumption (ng/kg bw per week).

Consumer Group		PFHpA (Min)	PFHpA (Max)	PFHxA	PFHxS	PFOA	PFOS	PFTrDA (Min)	PFTrDA (Max)	PFUnDA	PFHpA (Min)	PFHpA (Max)	PFUnDA	Cumulative EWI (Min)	Cumulative EWI (Max)
Toddlers	PEQ-EWI	0.00	0.03	0.00	0.00	0.63	3.15	0.00	0.02	0.02	0.00	0.03	0.00	3.80	3.85
	%TWI	0%	1%	0%	0%	14%	72%	0%	1%	0%	0%	1%	0%	86%	88%
	CB-%TWI *	1%		1%	0%	14%	36%	0%		0%	0%		0%	52%	
Adolescents	PEQ-EWI	0.00	0.01	0.00	0.00	0.26	1.33	0.00	0.00	0.01	0.00	0.01	0.00	1.61	1.63
	%TWI	0%	0%	0%	0%	6%	30%	0%	0%	0%	0%	0%	0%	37%	37%
	CB-%TWI *	0%		0%	0%	6%	15%	0%		0%	0%		0%	22%	
Adults	PEQ-EWI	0.00	0.01	0.00	0.00	0.20	1.02	0.00	0.00	0.01	0.00	0.01	0.00	1.23	1.24
	%TWI	0%	0%	0%	0%	5%	23%	0%	0%	0%	0%	0%	0%	28%	28%
	CB-%TWI *	0%		0%	0%	5%	12%	0%		0%	0%		0%	17%	
Elderly	PEQ-EWI	0.00	0.01	0.00	0.00	0.19	0.96	0.00	0.00	0.01	0.00	0.01	0.00	1.16	1.18
	%TWI	0%	0%	0%	0%	4%	22%	0%	0%	0%	0%	0%	0%	26%	27%
	CB-%TWI *	0%		0%	0%	4%	11%	0%		0%	0%		0%	16%	

* Value representing the % ratio between CB-EWI and the TWI.

**Table 6:** PEQ estimated intakes through fish and seafood consumption (ng/kg bw per week).

Consumer Group		PFBS	PFDA (Min)	PFDA (Max)	PFDS	PFDoDA	PFHpA (Min)	PFHpA (Max)	PFHxA	PFHxS	PFNA	PFOA	PFOS	PFPeA	PFTeDA	PFTrDA (Min)	PFTrDA (Max)	PFUnDA	Cumulative EWI (Min)	Cumulative EWI (Max)
Toddlers	PEQ-EWI	0.00	1.93	4.83	0.00	0.35	0.00	0.04	0.00	0.01	2.56	1.38	15.59	0.02	0.00	0.04	0.41	0.73	22.62	25.93
	%TWI	0%	44%	110%	0%	8%	0%	1%	0%	0%	58%	31%	354%	0%	0%	1%	9%	17%	514%	589%
	CB-%TWI *	1%	11%		0%	3%	1%		9%	0%	6%	31%	177%	13%	0%	3%		4%	259%	
Adolescents	PEQ-EWI	0.00	1.09	2.72	0.00	0.20	0.00	0.00	0.00	0.01	1.44	0.77	8.78	0.01	0.00	0.02	0.23	0.41	12.74	14.57
	%TWI	0%	25%	62%	0%	5%	0%	0%	0%	0%	33%	18%	199%	0%	0%	1%	5%	9%	289%	331%
	CB-%TWI *	0%	6%		0%	2%	0%		5%	0%	3%	18%	100%	7%	0%	2%		2%	146%	
Adults	PEQ-EWI	0.00	0.96	2.39	0.00	0.18	0.00	0.02	0.00	0.01	1.27	0.68	7.72	0.01	0.00	0.02	0.20	0.36	11.20	12.83
	%TWI	0%	22%	54%	0%	4%	0%	0%	0%	0%	29%	15%	175%	0%	0%	0%	5%	8%	255%	292%
	CB-%TWI *	0%	5%		0%	1%	0%		4%	0%	0%	15%	88%	7%	0%	2%		2%	128%	
Elderly	PEQ-EWI	0.00	1.03	2.58	0.00	0.19	0.00	0.02	0.00	0.01	1.37	0.73	8.31	0.01	0.00	0.02	0.22	0.39	12.06	13.83
	%TWI	0%	23%	59%	0%	4%	0%	0%	0%	0%	31%	17%	189%	0%	0%	0%	5%	9%	274%	314%
	CB-%TWI *	0%	6%		0%	1%	0%		5%	0%	3%	17%	94%	7%	0%	2%		2%	138%	

* Value representing the % ratio between CB-EWI and the TWI.

**Table 7:** Dietary PEQ and CB Exposure Assessment from Animal-based foods across Consumer Groups (ng/kg bw per week).

FC	PFBA	PFBS	PFDA (Min)	PFDA (Max)	PFDS	PFDoDA	PFHpA (Min)	PFHpA (Max)	PFHpS (Min)	PFHpS (Max)	PFHxA	PFHxS	PFNA	PFOA	PFOS	PFPeA	PFTeDA	PFTrDA (Min)	PFTrDA (Max)	PFUnDA	Cumulative EWI (Min)	Cumulative EWI (Max)
Toddlers																						
PEQ-Total EWI	0.00	0.00	2.14	5.35	0.09	0.67	0.00	0.09	0.00	0.01	0.00	0.01	2.92	2.69	21.56	0.06	0.01	0.05	0.48	0.85	31.07	34.81
PEQ-HI																					7.06	7.91
CB-HI																					3.98	
Adolescents																						
PEQ-Total EWI	0.00	0.00	1.22	3.05	0.06	0.43	0.00	0.03	0.00	0.00	0.00	0.01	1.67	1.47	11.98	0.02	0.00	0.03	0.27	0.50	17.40	19.50
PEQ-HI																					3.95	4.43
CB-HI																					2.15	
Adults																						
PEQ-Total EWI	0.00	0.00	1.05	2.62	0.04	0.34	0.00	0.04	0.00	0.00	0.00	0.01	1.42	1.18	10.03	0.02	0.00	0.02	0.23	0.42	14.54	16.35
PEQ-HI																					3.30	3.72
CB-HI																					1.76	
Elderly																						
PEQ-Total EWI	0.00	0.00	1.11	2.78	0.04	0.33	0.00	0.04	0.00	0.00	0.00	0.01	1.50	1.19	10.40	0.02	0.00	0.02	0.24	0.44	15.06	16.99
PEQ-HI																					3.42	3.86
CB-HI																					1.82	

## Data Availability

The original contributions presented in this study are included in the article/Supplementary Material. Further inquiries can be directed to the corresponding authors.

## Supplementary Materials

The following supporting information can be downloaded at https://www.mdpi.com/article/10.3390/toxics13110931/s1: Table S1: Evaluation of PEQ Intake through animal-derived food consumption.

## Abbreviations

The following abbreviations are used in this manuscript:

BMD	Benchmark Dose
CB	Concentration-Based
ECHA	European Chemicals Agency
EPA	Environmental Protection Agency
EWI	Estimated Weekly Intake
FC	Food Category
FDA	U.S. Food and Drug Administration
HBGV	Health-Base Guidance Value
HI	Hazard Index
HQ	Hazard Quotient
IARC	International Agency For Research On Cancer
IC	Index Compound
LB	Lower Bound
LOD	Limit of Detection
LOQ	Limit of Quantification
ML	Machine Learning
MLs	Maximum Levels
NAMs	New Approach Methodologies
PEQ	PFOA Equivalent
PFAS	Per- And Polyfluoroalkyl Substances
PFBA	Perfluorobutanoic Acid
PFBS	Perfluorobutanesulphonic Acid
PFDA	Perfluorodecanoic Acid
PFDoDA	Perfluorododecanoic Acid
PFDS	Perfluorodecanesulfonic Acid
PFHpA	Perfluoroheptanoic Acid
PFHpS	Perfluoroheptanesulfonic acid
PFHxA	Perfluorohexanoic Acid
PFHXS	Perfluorohexanesulphonic Acid
PFNA	Perfluorononanoic Acid
PFOA	Perfluorooctanoic Acid
PFOS	Perfluorooctanesulfonic Acid
PFPeA	Perfluorinatedpentanoic Acid
PFTeDA	Perfluorotetradecanoic Acid
PFTrDA	Perfluorotetradecanoic Acid
PFUnDA	Perfluoroundecanoic Acid
QSAR	Quantitative–Structure Activity Relationship
REACH	Registration, Evaluation, Authorisation and Restriction of Chemicals
RPF	Relative Potency Factor
TEF	Toxic Equivalency Factor
TWI	Tolerable Weekly Intake
UB	Upper Bound
